# Evaluation of the impact of the voucher and accreditation approach on improving reproductive health behaviors and status in Kenya

**DOI:** 10.1186/1471-2458-11-177

**Published:** 2011-03-23

**Authors:** Charlotte Warren, Timothy Abuya, Francis Obare, Joseph Sunday, Rebecca Njue, Ian Askew, Ben Bellows

**Affiliations:** 1 Population Council, General Accident Insurance House, Ralph Bunche Road, PO Box 17643, Nairobi 00500, Kenya

**Keywords:** Vouchers, Out-put based approach

## Abstract

**Abtsract:**

**Background**

Alternatives to the traditional 'supply-side' approach to financing service delivery are being explored. These strategies are termed results-based finance, demand-side health financing or output-based aid which includes a range of interventions that channel government or donor subsidies to the user rather than the provider. Initial pilot assessments of reproductive health voucher programs suggest that, they can increase access and use, reducing inequities and enhancing program efficiency and service quality. However, there is a paucity of evidence describing how the programs function in different settings, for various reproductive health services. Population Council, funded by the Bill and Melinda Gates Foundation, intends to generate evidence around the 'voucher and accreditation' approaches to improving the reproductive health of low income women in Kenya.

**Methods/Design**

A quasi-experimental study will investigate the impact of the voucher approach on improving reproductive health behaviors, reproductive health status and reducing inequities at the population level; and assessing the effect of vouchers on increasing access to, and quality of, and reducing inequities in the use of selected reproductive health services. The study comprises of four populations: facilities, providers, women of reproductive health age using facilities and women and men who have been pregnant and/or used family planning within the previous 12 months. The study will be carried out in samples of health facilities - public, private and faith-based in: three districts; Kisumu, Kiambu, Kitui and two informal settlements in Nairobi which are accredited to provide maternal and newborn health and family planning services to women holding vouchers for the services; and compared with a matched sample of non-accredited facilities. Health facility assessments (HFA) will be conducted at two stages to track temporal changes in quality of care and utilization. Facility inventories, structured observations, and client exit interviews will be used to collect comparable data across facilities. Health providers will also be interviewed and observed providing care. A population survey of about 3000 respondents will also be carried out in areas where vouchers are distributed and similar locations where vouchers are not distributed.

## Background

Stagnating indicators for several reproductive and child health conditions in many countries of Africa and Asia are a major concern for governments and development partners striving to achieve the Millennium Development Goals (MDGs). These indicators, including maternal and infant morbidity and mortality, are poorest among low-income populations. Weak and inefficient health systems sustain these inequities in access to and use of essential services. There remains an over-reliance on financing the inputs of service delivery in the public sector, supported by the beliefs that: (i) service purchasing is too difficult, and (ii) that the private sector is not willing or able to serve low-income clients.

Recognizing that the cost of delivering reproductive health services to low-income populations will always require subsidization by the government and/or development partners, alternatives to the traditional 'input-based' approach to financing health service are being explored. Broadly termed results-based financing, demand-side financing (DSF) or output-based aid (OBA), these alternatives include a range of interventions that channel government or donor subsidies to the service user rather than the service provider [1,2]. The goal is to increase access to and use of key services by subsidizing the user to purchase the service, preferably by choosing a provider from among a number of competitive alternatives. This can stimulate competition between providers thereby motivating improvements in access to and quality of services. Providers who perform well by attracting users receive service payments that cover delivery costs and a profit margin.

An output-based approach, therefore, uses explicit performance-based subsidies to motivate providers to deliver selected reproductive health services at a specified level of quality and at an affordable cost so that the economically disadvantaged are not excluded. Several interventions are currently being developed and tested, including franchising and contracting, social health insurance, conditional cash transfers and vouchers. Voucher programs are intended to achieve a number of policy goals: (i) by reducing financial barriers, they can increase access to services generally, and reduce inequities by making them affordable to the poor and other underserved groups; (ii) by accrediting several providers to offer the service at the same price, they can increase choice for clients; (iii) by including more than one provider, competition for clients with vouchers can increase efficiency in delivery and possibly reduce prices further; and (iv) by requiring quality standards before accreditation, quality of care can be improved.

Vouchers for reproductive health are not new; Taiwan and Korea successfully used them in the 1960s to increase access to family planning [3] and Nicaragua implemented two voucher projects for sexually transmitted infections (STI) services in 1995. More recently there has been a resurgence of interest in vouchers. The German Development Bank (KfW) is currently supporting OBA programs for reproductive health in collaboration with government and non-government partners in Cambodia, Kenya and Uganda, is planning to start a similar program in Tanzania and supports the sector wide approach funded voucher program in Bangladesh. These programs will pilot-test voucher schemes for deliveries assisted by skilled personnel and family planning, with additional country specific services including prevention and management of STIs, care for survivors of sexual assault and safe abortion.

In brief, these programs generally establish a voucher management agency (VMA) that produces and distributes subsidized vouchers to clients, who purchase or receive for free a voucher for a specific service at a price that has been determined to be affordable for the lowest-income clients. The VMA invites a number of service providers (individually or within an organization, which can be public, non-profit or for-profit) to participate in the program. Ideally providers must demonstrate that they are able to provide the services at a specified standard of quality of care. They are then accredited to participate subject to periodic quality reviews. The client can redeem the voucher for the specified service at the accredited providers. Following the visit, the provider is reimbursed upon submission of supporting documentation to the VMA. The reimbursement could be fee-for-service, capitated payment, diagnostic related group payment or other common form.

Initial findings from the few assessments of reproductive health voucher programs suggest that, if implemented well, they have the potential to achieve the policy objectives of increasing access and use, reducing inequities and enhancing program efficiency and service quality [4,5]. There is, however, a paucity of evidence describing how: (1) the voucher programs function in different settings, for various reproductive health services delivered through public, for-profit or non-profit organizations; and (2) the voucher program affects the operational efficiency and business model used by service delivery organizations and individual providers. There is limited understanding of their effect on the quality of care received by clients and on levels of service utilization, especially among the poor and underserved. There is also limited evidence to date on their impact on RH behaviors and status at the individual and population levels, especially on those health status indicators relevant for the MDGs.

### Output Based Aid Voucher Program in Kenya

The voucher program in Kenya is funded by KfW. It was overseen by the National Coordinating Agency for Population and Development (NCAPD) from 2006-2011 when it moved to the Ministry of Public Health and Sanitation. It focuses on subsidizing comprehensive Safe Motherhood (SM) services and long term family planning (FP) methods to economically disadvantaged clients in Kisumu, Kitui and Kiambu districts, and Korogocho and Viwandani informal settlements in Nairobi. Additional vouchers were made freely available for women seeking gender-based violence (GBV) services.

*Phase I *of the current voucher project in Kenya took place between 2006 and 2009 in 54 accredited facilities: 18 in Kisumu District, 17 in Kiambu District, 12 in Nairobi and seven in Kitui District. The key objective was to significantly reduce maternal and neonatal mortality by increasing the number of deliveries at health facilities and improve access to appropriate health services for the poor through incentives for increased demand and improved service provision.

*Phase II *is currently underway until November 2011. The VMA continues to assess and disburse funds in the existing facilities but technical assistance is being sought to maintain the scaling up of the project and support the new program management unit in the Ministries of Health.

## Methods/Design

### Hypotheses to be tested

i). At Facility Level

a) Accredited facilities will have a greater increase in average utilization of essential Maternal and New born health (MNH) care and FP services compared to control facilities between baseline and follow-up surveys.

b) Accredited facilities will have a greater increase in the proportion of poor clients for essential MNH care and FP services compared to control facilities between baseline and follow-up surveys. (Poverty is measured using three indices: participatory scale, standard household assets scale and a food insecurity scale)

c) The quality of essential MNH and FP services in voucher facilities will be equal to or greater than the quality of the same in non-accredited facilities.

ii). At Population Level:

a) Communities served by voucher distributors for MNH and FP services will have greater increase in the proportion of facility-based births compared to the comparison communities at baseline and follow up surveys

b) Communities served by voucher distributors for MNH and FP services will have greater increase in the proportion of facility-based births among the poor compared to the poor in comparison communities at baseline and follow up surveys.

### Study Objectives

This study aims to evaluate the impact and effectiveness of the voucher and accreditation approach in Kenya.

### Specific Objectives

1. To assess the effect of the voucher and accreditation approach on increasing access to, quality of, and reducing inequities in the use of, selected RH services

2. To evaluate the impact of the voucher and accreditation approach on improving reproductive health behaviors and RH status and reducing the inequities at the population level.

### Study design

The study will employ a before and after quasi-experimental design with a control group where surveys will be undertaken among the target population for the voucher program before and after its introduction and also among an equivalent comparison population living in areas not served by a voucher program in order to control for potential time dependent confounding.

In order to address the first objective, facilities will be the primary sampling unit to measure access to and quality of care and service statistics. Health facility assessments, including providers' technical competence, skills and time-utilization, and clients' perceptions of quality of care at specified intervals at accredited and non-accredited facilities will be undertaken. The district-level administrative unit will be used to generate clusters of heath facilities that are accredited and those that are not. These two sets of facilities will be in the same or similar districts to maximize the likelihood of the populations having similar social, cultural, economic characteristics, and having similar RH behaviors among women aged 15 to 45 years and among pregnant women. As some degree of variability is expected between the districts in terms of the background characteristics mentioned above, four districts will be selected. By the end of Phase I, there were 54 facilities accredited in Kenya. Five of these will be randomly selected from each district and Nairobi making a total of 20 facilities that will then be matched with non-accredited facilities in similar nearby districts.

Given that the accredited facilities will self-select to the experimental group through choosing to participate in the voucher program, there is a strong likelihood that they will be different from those not choosing or not invited to participate. We cannot predict *a priori *how they might be different, for example, perhaps the contracted providers are more entrepreneurial or perhaps more socially motivated. To maximize the equivalence of these groups, thereby enhancing the validity of the design, a sampling design known as pair-wise matching will be used. In this design, the characteristics of interest (those characteristics that may influence a provider's or facility's performance above and beyond their use of the voucher and accreditation model) are measured for each accredited provider in the experimental sample upon recruitment, and a profile established for each provider. Researchers then identify 'equivalent' non-accredited providers for the control group. Examples of these types of characteristics include type of practice, professional skills mix, profile of clientele, location, and fees charged, among others.

The second objective of this research is to conduct population-level surveys among representative samples of women, men and adolescents stratified by socio-economic status in geographic areas served by accredited and non- accredited facilities in the selected districts. The sample size is based on the national proportion of facility-based births; 42% of all births. We assume the national figure is representative of the proportion of facility-based births in the voucher region. To detect a 14% increase in the proportion of facility-based births, we will need 1078 experimental subjects and 1078 control subjects to be able to reject the null hypothesis that the proportion of facility-based births for experimental and control subjects are equal with probability (power) 0.8. The Type I error probability associated with this test of the null hypothesis is 0.05. We will use an uncorrected chi-squared statistic to evaluate this null hypothesis. The survey will measure indicators described in table 1. The expected results include intervention-dependent RH outcomes (pregnancy-and birth-related complications, unintended pregnancy, inter-birth intervals, reported STI treatment, among others); RH-related care behaviors (antenatal care, ANC; skilled delivery; postnatal care; lactational amenorrhea, LAM, breastfeeding; contraceptive use); awareness of RH issues, use of services, out of pocket spending, and expectations for use of services.

**Table 1:** Broad indicators for the assessment of OBA project in Kenya

Areas of focus	Indicators
**Knowledge**	Provider competence; patient recognition of signs and symptoms of illness

**Utilization**	RH service utilization, client load, client socio-economic profile, and market share for voucher and accreditation services; Proportion of RH services provided by accredited and non-accredited facilities at district level, by public and non-public sector

**Targeting**	Proportion of eligible people who received voucher; percent of voucher holders who meet poverty scores

**Quality**	RH service quality as measured by facility readiness; provider competence; information provision; compliance with norms; follow-up support; client perceptions; among others

**Costs**	out-of-pocket expenses; facility revenue and expenses on voucher services include willingness to pay

**Disease burden and health status**	proportion of complicated pregnancies; respondents' socio-demographic characteristics, health-seeking behaviors by health condition, RH conditions and behaviors relevant to the service being evaluated, experiences and perceptions of RH services received; measures of pregnancy and birth-related complications, unintended pregnancies, inter-birth intervals, reports of STI symptoms, contraceptive, and condom, lactational amenorrhea method (LAM) use, and attendance for antenatal, delivery and postnatal services

### Data collection procedures

#### a). Health Facility Assessments

Population Council will conduct two health facility assessments [6] to assess quality of care provided in public, faith-based and private study facilities. An initial assessment will be undertaken in both accredited and non-accredited facilities to determine the comparability of the facilities and to provide baseline measures of the quality of care. To determine the sustainability of the quality of care provided an additional assessment will be undertaken at 12-15 months later to determine the extent to which the quality of care has changed. In addition, data collected through routine monitoring of service statistics will provide further information about client load, services mix, and client characteristics. Data collection procedures for each component of these assessments are as follows:

##### i) Facility Inventory

An inventory of available resources including facility infrastructure, staffing numbers and skills mix, services provided, staff training undertaken, availability of equipment, commodities, test kits, stationary (client cards and notes), medications required to provide the services within the intervention will be undertaken. The head of the facility will be approached by a nurse/midwife researcher. The researcher will request the in-charge to guide them around the facility to observe and record all relevant information on a checklist.

##### ii) Review of service statistics

Statistics related to routine program data on utilization of MNH/family planning services for a 6 or 12 month period prior to the assessment visit will be collected. We will also record the number of new and continuing clients coming to a clinic for MNH/FP services as well as other health services. Monthly trends in the numbers of new and continuing ANC, post-natal care (PNC) and family planning clients as well as for other services will be obtained from facility records.

##### iii) Interviews with healthcare providers

All providers at 40 MNH-FP units will be approached for interview. Eligible providers available on the day of data collection at the facility will be interviewed concerning MNH and FP services. Interviews will ascertain their perceptions of barriers and operational challenges that may influence voucher clients' acceptance of services and the provider's attitudes towards the accreditation process. In addition there will be assessment of provider knowledge and skills for MNH and FP and other related SRH services, as well as their understanding of the organizational setup and description of related activities. It is expected that four to eight providers will be eligible to participate in the hospitals and between two and four at the health centers and dispensaries. This will give around 80 providers in each group (Total 160).

##### iv) Observations of Client-Provider Interactions

To assess the quality of care at each facility, all providers participating in the facility evaluation and who deliver the MNH/FP services will be asked for permission to observe their consultations. Recruitment of providers will be done following sensitization meetings held with the district health management teams (or equivalent). Researchers will hold group meetings with the management and healthcare providers in each participating facility to introduce the purpose and methods of the study and to request their participation. The tools will also be pre-tested among a small group of women with similar characteristics as the study population to identify potentially negative consequences and modified accordingly.

Observations of client-provider interactions (CPI) will be conducted during FP visit, labor, pre-discharge from the maternity unit and delivery, six weeks postpartum. The CPI encompasses both the process (how clients are treated and whether they actively participate) and the content (what they are told, technical competence, accuracy of information, provision of essential information) of a consultation. After obtaining informed consent from the client, a structured non-participatory observation of the client-provider interaction will be undertaken to determine the quality of care provided. Subsequent sessions for which consent has been received will be observed until 6 randomly selected antenatal, delivery, PNC and FP clients in each facility have been observed. This includes government, non-governmental, faith-based and private facilities. We acknowledge that observing client provider interaction may bias in a positive direction the results obtained on quality of care. We will be spending more than one day at each site, so that the presence of the research team becomes more familiar and the behavior of the providers becomes more normative.

Samples of clients attending each type of consultation will be recruited if they meet the following eligibility criteria: are accessing family planning or maternity care including postnatal care for themselves (and/or their babies) at delivery or one of the pre-discharge, one-week or six-week postpartum consultation times; are aged over 18 years (the small proportion of clients that are less than 18 years will not justify the difficulties in obtaining parental/guardian permission); are aged below 45 years (the small proportion of women giving birth/accessing FP above this age will be excluded); give their informed consent for their consultation to be observed and the key actions taken recorded, and to be interviewed on exiting from the consultation. All women satisfying these inclusion criteria will be recruited until the required sample sizes have been reached.

##### v).Client exit interview

Exit interviews will be held with each client who was observed by a trained interviewer to ascertain their perceptions of the service received. The client will be introduced to the interviewer following the CPI. To measure the magnitude of changes in the quality of services provided, composite summary scores will be developed for a series of key indicators by aggregating the mean scores of key items being assessed for each individual client-provider interaction being observed. This scoring system will categorize whether an accepted standard of quality has been met. For each study group, a mean score per group will be calculated for each indicator and for the composite summary score to enable statistical comparisons to be made between experimental and comparison groups over time. Examples of the types of individual items and key indicators are presented in Table 2.

**Table 2:** Examples of indicators to make composite scores of quality of care

**Quality of**:	**Observed provider actions**:
a. Client - provider rapport (0-7)	Client greeted warmly, Discussed medical conditions, Asked if client understood information, Encouraged client to ask questions, Used client's name, Help in decision-making, Consultation time > 15 minutes

b. FP method counseling (0-6)	Discuss reproductive intentions, discuss previous use of FP, Discuss 2 or more methods, provide choice regarding preferred method, discuss how chosen method works, explain (dis)/advantages of chosen method

c. ANC counseling	Birth planning, danger signs, infant feeding, fertility intentions

d. PNC counseling on danger signs since childbirth (0-10)	Ask about: bleeding since birth, color/smell of vaginal discharge, condition of perineum/CS scar, fever, headache or blurred vision, swelling in face, hands or feet, signs of thrombophlebitis, tiredness or breathlessness, convulsions or fits

#### b). Population Survey

Population Council will conduct baseline and end-line population level surveys with a randomly selected sample of men and women aged 15-45 years from the catchment communities of all study facilities and have had a pregnancy or a pregnant partner during the last 12 months or started a new FP method. Adolescents 15 - 17 years will only be interviewed following parental consent. Surveys will compare patterns of service use and perception and to compare any differences between communities that have ready access to the voucher and communities that do not have access.

Facility catchment areas will be identified as either "experiment" or "control" based on the presence of an accredited facility. A complete list of villages (administratively referred to as "sub-locations") in the catchment areas will be made and a sample taken from areas with and without an accredited facility. In each selected village, teams will randomly select a seed household and then identify every third household along village roads. At each of these "core" households, teams will inquire about pregnancies in the core household and in the two adjacent households. In this way, teams will identify and visit households in which a pregnancy or recent delivery is reported.

Community members will be asked standardized, and sex-specific, questions on access and use of services, attitudes, experiences and reasons for service use/non-use of voucher and RH issues. This will offer a comparison between voucher holders *vs*. non-voucher holders, as well as offer insights into preferences for the accredited services and reasons for use/non-use of these. Enumerators will be trained on proper technique and ethical conduct. Training of research assistants is likely to take a minimum of eight days including a pretest in the field. Table 3 describes examples of operational results and indicators to compare accredited and non- accredited facilities and communities. Paper questionnaires and portable digital assistants (PDAs) will be used to capture quantitative data. Data from paper questionnaires will be keyed into Epidata 3.1 and exported into Stata 10 for analysis. Data from PDAs will downloaded into an MS Access database before being exported into Stata 10 for analysis.

**Table 3:** Examples of operational results and indicators to be used to compare results from the accredited and non-accredited health facilities and communities

1. to assess the effect of the voucher program on increasing access to, quality of, and reducing inequities		
**Results**	**Indicators**	**Data source**

Provision of services reported as acceptable by providers and clients	Clients received comprehensive ANC and PNC	Client exit
	Clients referred for complicated deliveries	Provider interview
	Clients referred for other services	Population survey

Increase in clients using MNH/FP services including poor women	% clients accessing different service by socio-economic status	Service statistics Client exit & population survey

Increase in FP clients accepting long term methods	% clients using LAPM	Service statistics
		Client exit & population survey

Improved attitudes of service providers towards poor women	% Providers indicating non discriminatory attitudes	Provider interview
	% Clients recommending services to others	Population survey

**2 Evaluate the impact of voucher program on RH behaviors, status and reducing inequities**		

**Results**	**Indicators**	**Data source**

Reduced incidence of unintended pregnancies	% women who become pregnant/%planned pregnancy	Client Exit
	% clients with correct knowledge of fertile period	Population survey

Increased duration of contraceptive use among all women and poor women	Among all respondents and subgroups of poor:	Population survey
	• Ever/Current use of FP method	
	• Discontinuation FP rates in 12 months	
	• Ability to achieve fertility goals	

Decreased stigmatization at community level of poor women	Perceived barriers to accessing services: costs, distance, quality, waiting times, stigma surrounding service	Population survey

#### c).Qualitative study

In order to enhance the findings from population surveys and address unforeseen questions arising from other components of the study, in-depth interviews (IDIs) and focus group discussions (FGDs) with healthcare providers and key informants will be conducted. These interviews will be used to gain a deeper understanding into the motivations, perceptions, and priorities of the healthcare providers regarding voucher and accreditation. The provider IDIs and FGDs will focus more specifically on: services offered; attitudes towards voucher and accreditation, including effects on workload; benefits and challenges of the voucher and accreditation; perception of clients' views; the referral system and other healthcare needs. Before the FGDs and IDIs, participants will be provided with any necessary information to complete their understanding of the nature of the research. The researcher will discuss with the participants their experience with the research in order to monitor any unforeseen negative effects or misconceptions.

FGDs will be carried out with groups of male and female voucher and non-voucher users (aged 18 years and over) as well as with providers. These will take place alongside the baseline and endline surveys, as well as on an ad hoc basis when needed during the project. These will be used to gain a deeper understanding into the motivations, perceptions, and priorities of the local community regarding vouchers and service use. The FGDs will address the following broad themes: motivations for healthcare use and selection/use of the RH services; attitudes towards voucher and accreditation; communication/interaction with different providers; contraceptive and sexual health behavior, including communication with partners and other community members about RH services. All participants in the FGDs will be requested to respect confidentiality and to agree to not to divulge any information heard during the discussion outside of the group.

FGDs of one to two hours will be held in four randomly selected populations within the surveyed districts:

1 FGD: 6-8 younger women who are currently or have been voucher users (< 25 years)

1 FGD: 6-8 older women who are currently or have been voucher users (25 years & over)

1 FGD: 6-8 younger women who have never used voucher (< 25 years)

1 FGD: 6-8 older women who are have never used vouchers (25 years & over)

1 FGD: 6-8 younger men who/or partners are currently or have been voucher users (< 25 years)

1 FGD: 6-8 older men who are currently or have been voucher users (25 years & over)

1 FGD: 6-8 younger men who/or partners who have never used voucher (< 25 years)

1 FGD: 6-8 older men who/partners who have never used vouchers (> 25 years)

### Data Management and Analysis

The Data Management Unit in Population Council will store all data in password protected computers. Hard copies of questionnaires, anonymised transcriptions and tapes of the group discussions will be stored securely in a locked cabinet, in accordance with the Population Council policy and the Kenya Data Protection Policy.

Analyses of facility data will be undertaken and the proportion of women receiving an acceptable quality of service will be calculated. The methodology to calculate the proportion of women receiving an acceptable quality is similar to the Lot Quality Assurance Sampling (LQAS) approach that has been used in Kenya and elsewhere for assessing quality [7]. LQAS follows the principle that an entire group (lot) of services is deemed poor quality if a certain proportion within a small sample does not reach a minimum standard. LQAS applies cumulative probabilities calculated with a binomial formula to select small sample sizes and decision criteria for judging a group of providers.

In addition, time series analyses will be conducted in order to estimate: mean monthly number of clients obtaining RH services, by type, at accredited and non-accredited providers; mean monthly number and proportion of clients in the lowest economic quintile obtaining RH services, by type, at accredited and non-accredited facilities and proportion of voucher services among all RH services at accredited facilities.

Population-level surveys provide the opportunity to measure reproductive health indicators, including both reported health status, behaviors and healthcare utilization, among populations being served by a voucher program and comparable populations not served by the voucher program. Statistical comparisons between these indicators can then be used to detect any differences between the populations at 1% and 5% level of significance. We will also compare concentration index scores for selected RH indicators calculated from the data collected among accredited and non-accredited populations. The concentration index is a widely used indicator for quantifying the degree of income related inequality in a specific health indicator and will be used to provide evidence of the extent to which voucher and accreditation approach reduces inequities. Qualitative data will be captured on paper and audio tapes and later transcribed into MS Word before exporting into QSR Nvivo 8 for analysis using thematic framework.

## Discussion

### Ethical issues

Informed consent will be obtained separately for each interview. For all the tools, provisions will be made to train researchers to ensure that guidance on ethical conduct is clearly understood and implemented. Such training will include sessions and exercises regarding the meaning and process of informed consent, the importance of protecting the privacy of subjects, and confidentiality of the information obtained from them. The research team will also be trained to listen and observe intently without displaying any judgmental attitude towards information they receive from the informants and on other critical ethical issues. The research teams will discuss and develop measures in relation to data recording style, personal identifiers, transcription and processing procedures, lifespan of unprocessed data, type and places of storage, and data safety and right of access.

All interviews will only be recorded after obtaining written informed consent from the interviewee. From the outset, it will be made clear to participants that they have a right to withdraw from the research at any time. At the end of the interview, participants will be provided with any necessary information to complete their understanding of the nature of the research. The researcher will discuss with the participants their experience with the research in order to monitor any unforeseen negative effects or misconceptions.

No immediate tangible benefit is likely to accrue to the subjects through their participation and this will be made clear when obtaining informed consent. However, the potential benefits to healthcare services and the women who use them will be described to potential participants, so that they are fully aware that the data gathered will be used to provide recommendations to the Kenya Ministries of Health, as well as to healthcare providers and communities in other countries. If any informant reports problems that will require medical and or psychosocial attention, they will be referred to existing services.

If the researchers find out information or observe activities during site visits that reflect poorly on the quality of health services provided or the health system in general, this will be recorded and incorporated into a summary report. The intervention team will try to address these issues (e.g. training of healthcare providers will include interpersonal skills as well as clinical skills). If, during a client/provider interaction, a client is perceived to be at medical risk, the observer (who will be medically trained) will be trained to intervene. Given the sensitive nature of the information to be gathered, protecting and respecting the confidentiality and privacy of informants will be a critical consideration throughout the study.

All participants will receive the following information:

• Aim of the study and methods to be used

• Institutional affiliations of the research

• Anticipated benefits and potential risks and follow-up of the study

• Discomfort it may cause

• Sensitive questions regarding sexual behavior, partners and condom use will be asked, though they may choose not to answer any questions

• Questionnaire administration will increase time at clinic

• Right to abstain from participating in the study, or to withdraw from it at any time, without reprisal

• Measures to ensure confidentiality of information provided

• Study numbers will be used on questionnaires to maintain anonymity of study participants

• No information will be divulged to partners or other third parties

• Monetary compensation will only be provided if participant has to travel for the interview

• Contact details of the study coordinator for any questions or concerns

During health facility assessments, informed consent will be obtained jointly for the observation and exit interview. During the recruitment interviews at the facilities, women will be asked a number of potentially sensitive questions, including their HIV status and their reproductive behavior and perceptions of contraceptive use. To avoid the risk of others overhearing this information, interviews will be conducted in strictly private settings with and ample time to ensure that privacy and confidentiality can be guaranteed.

During the provider interviews, respondents will be assured that no-one, including their supervisor, will know what they say and their names will not appear on the questionnaire. However, anonymised summary information and opinions from providers will be presented as part of the summary report. Results of client and provider interviews will be presented in reports in an aggregated manner such that responses cannot be traced back to individuals. Population Council staff will visit the data collection site to ensure interviewers adhere to confidentiality procedures.

During the population survey, written consent will be administered to all respondents. According to Kenyan law, the age of consent for participating in research is 18 years. Given that it would be difficult to trace parents of adolescents attending FP clinics no one will be interviewed under 18 years of age. However for the community survey any adolescents aged 15-17 years will be interviewed at the household level only if an adolescent assents and parental consent has been obtained first.

Names will only be recorded for women agreeing to any follow-up visits to facilitate contact. This information, together with their contact information, will be recorded on a separate sheet and kept physically separate from the data collection instruments containing their information, and will only be linked to questionnaires by study numbers. Only the research team coordinator will have access to both the names and instruments. During follow-up interviews, a woman may not want others to know that she is participating in the study, and all women will be offered the opportunity to hold the interview in a location of her choosing, with travel costs reimbursed to ensure that this is a viable option. The majority of clients will be interviewed at the clinic or during community surveys. However if/where the informants are requested to travel specifically for interview, the study will compensate informants (at an average rate of US $5 per study participant) for any inconvenience. Researchers conducting FGDs will be reminded how to preserve confidentiality and ask the groups to respect personal information before discussions start.

### Ethical clearance

The research protocol has been reviewed by key stakeholders and ethical clearance has been granted by the Kenya Medical Research Institute (KEMRI) Ethical Review Board and the Population Council institutional review board (IRB).

## List of abbreviations

BMGF: Bill and Melinda Gates Foundation; CIDA: Canadian International Development Agency; CPI-Client: Provider Interaction; DfID: Department for International Development (UK); DSF: Demand Side Financing; FGD-Focus Group Discussion; FP: Family Planning; HFA-Health Facility Assessment; KEMRI-Kenya Medical Research Institute; KfW: Kreditanstaldt fuer Wiederaufbau (German Development Bank); LAM-Lactational Amenorrhea Method; LQA: Lots Quality Assurance; MNH: Maternal and Child Health; MDGs: Millennium Development Goals; MIS: Management Information System; MNH: Maternal and Newborn Health; NCAPD: National Coordinating Agency on Population and Development (Kenya); OBA: Output-Based Aid; STI: Sexually Transmitted Infection; RH: Reproductive Health; V & A: voucher and accreditation programs; VMA: Voucher management agency.

## Competing interests

The authors declare that they have no competing interests

## Authors' contributions

CW, IA and BB were involved in the conceptual design of the study. TA and BB were involved in drafting, re-organizing and overall revision of the manuscript. FO, JS and RN were involved in the revision of the manuscript. All the authors are involved in the implementation of the project and have read and approved the final manuscript.

## Pre-publication history

The pre-publication history for this paper can be accessed here:

http://www.biomedcentral.com/1471-2458/11/177/prepub

## References

[B1] Janisch CP Potts M Smart aid--the role of output-based assistance Lancet 2005 366 9494 1343 4 10.1016/S0140-6736(05)67547-2 16226600

[B2] Gorter A Sandiford P Rojas Z Salvetto M Competitive voucher schemes for health background paper 2003 Instituto Centro Americano de la Salud (ICAS)

[B3] Cernada G Chow LP The coupon system in an ongoing family planning program Am J Public Health Nations Health 1969 59 12 2199 208 10.2105/AJPH.59.12.2199 5389504 PMC1226800

[B4] Meuwissen LE Gorter AC Knottnerus AJ Impact of accessible sexual and reproductive health care on poor and underserved adolescents in Managua, Nicaragua: a quasi-experimental intervention study J Adolesc Health 2006 38 1 56 10.1016/j.jadohealth.2005.01.009 16387251

[B5] Bhat R Mavalankar DV Singh PV Singh N Maternal healthcare financing: Gujarat's Chiranjeevi Scheme and its beneficiaries J Health Popul Nutr 2009 27 2 249 58 10.3329/jhpn.v27i2.3367 19489419 PMC2761781

[B6] HFA TWG Profiles of Health Facility Assessment Methods, MEASURE Evaluation, JSI, USA 2007

[B7] Valadez JJ Transgrud R Mbugua M Smith T Assessing family planning service-delivery skills in Kenya Stud Fam Plann 1997 28 2 143 50 10.2307/2138116 9216034

